# Sesamol: A Natural Phenolic Compound with Promising Anticandidal Potential

**DOI:** 10.1155/2014/895193

**Published:** 2014-12-09

**Authors:** Moiz A. Ansari, Zeeshan Fatima, Saif Hameed

**Affiliations:** Amity Institute of Biotechnology, Amity University, Manesar, Gurgaon, Haryana 122413, India

## Abstract

We investigated the antifungal effects of sesamol (Ses), a natural phenolic compound, and exemplified that it could be mediated through disruption of calcineurin signaling pathway in *C. albicans*, a human fungal pathogen. The repertoire of antifungal activity not only was limited to *C. albicans* and its six clinical isolates tested but also was against non-*albicans* species of *Candida*. Interestingly, the antifungal effect of Ses affects neither the MDR efflux transporter activity nor passive diffusion of drug. We found that *C. albicans* treated with Ses copies the phenotype displayed by cells having defect in calcineurin signaling leading to sensitivity against alkaline pH, ionic, membrane, salinity, endoplasmic reticulum, and serum stresses but remained resistant to thermal stress. Furthermore, the ergosterol levels were significantly decreased by 63% confirming membrane perturbations in response to Ses as also visualized through transmission electron micrographs. Despite the fact that Ses treatment mimics the phenotype of compromised calcineurin signaling, it was independent of cell wall integrity pathway as revealed by spot assays and the scanning electron micrographs. Taken together, the data procured from this study clearly ascertains that Ses is an effectual antifungal agent that could be competently employed in treating *Candida* infections.

## 1. Introduction

*Candida albicans*, an opportunistic human fungal pathogen, is the most common cause of the invasive fungal diseases [1]. It resides normally within the host body but during the immunosuppressed or immunocompromised conditions like AIDS, cancer, and organ transplant; it can cause several diseases such as oral thrush, vulvovaginitis, esophagitis, and cutaneous infections. It is also among the most common causative agents for nosocomial infections in patients who have undergone surgery or organ transplantation, have diabetes, and take excessive antibiotics and are neutropenic [2, 3]. Some of the most common isolates from candidiasis and candidemia are* C. albicans, Candida glabrata, Candida krusei, Candida tropicalis, *and* Candida parapsilosis* [4]. Among all* Candida* species* C. albicans* is the most prominent causative agent of the diseases [5]. Due to daily augmentation in cases of the patients suffering from the diseases caused by* Candida* species, it has become unavoidable to find the cure for this evader.

Present treatment regime includes several classes of antifungal which are in use to treat the infection caused by the fungal pathogens. For instance azoles, polyenes and allylamines target ergosterol pathway. Echinocandins and pyrimidines target cell wall and nucleic acid synthesis respectively [6]. Due to excessive usage of the current therapeutic drugs, there occur several quandaries associated with these drugs. Multidrug resistance (MDR), severe side effects, high cost, and lesser efficiency are some of the most renowned and sufficient causes for the scientific community to take the initiative steps for finding the newer drugs having lesser toxicity and targeting the new pathways. Natural compounds due to their cost effectiveness and lesser toxicity could prove to be a better option for the new era of the antifungal drug with new targets and better activity.

Sesamol (Ses) (3,4-methylenedioxyphenol) is a well-known antioxidant which is extracted from the sesame oil from* Sesamum* species [7]. There are several beneficial effects known for sesamol that have been reported like antioxidant, chemoprevention, antimutagenic, and antihepatotoxic activities and induction of apoptosis of cancer and cardiovascular cells [8]. In this study, we deciphered the antifungal activity of Ses against human fungal pathogen* C. albicans* as well as various non-*albicans* species. We found that antifungal action of Ses may be linked with hindered calcineurin signaling pathway and disruption of membrane homeostasis. Considering the significance of ergosterol as the target of known antifungal like azoles, Ses could well be used as adjunct to known drugs for better antifungal therapy.

## 2. Materials and Methods

All media chemicals YEPD (yeast extract peptone dextrose), agar, rhodamine 6G (R6G), 2-deoxy-D-glucose (2-DOG), 2,4-dinitrophenol (2,4 DNP), and* n*-heptane, was purchased from HiMedia (Mumbai, India). Sodium chloride (NaCl), calcium chloride (CaCl_2_), lithium chloride (LiCl), potassium chloride (KCl), mannitol, disodium hydrogen orthophosphate, potassium dihydrogen orthophosphate, dipotassium hydrogen orthophosphate, sodium hydroxide, D-glucose, and sodium dodecyl sulphate (SDS) were obtained from Fischer Scientific. Calcofluor white (CFW), Congo red (CR), and sesamol (Ses) were obtained from Sigma Chemical Co. (St. Louis, MO, USA).

### 2.1. Growth Media and Strains Used

The reference strains of* C. albicans* used in this study were ATCC 10261 and ATCC 24433. The clinical isolate strains of* C. albicans *were D1, D2, D4, D7, D18, and D20 and non-*albicans* species include ATCC 90030 (*Candida glabrata*), D9 (*Candida tropicalis*), D11 (*Candida parapsilosis*), and D46 (*Candida krusei*). All the strains of* Candida albicans* were cultured in YEPD broth with the composition of yeast extract 1% (w/v), peptone 2% (w/v), and dextrose 2% (w/v). For agar plates 2% (w/v) agar (HiMedia, Mumbai, India) was added to the media. All* Candida *strains were stored in 30% (v/v) glycerol stock at −80°C. The cells were freshly revived on YEPD broth and transferred to agar plate. The cells were grown at 30°C on agar plate before each study to ensure the revival of the strains.

### 2.2. Drug Susceptibility Testing

Drug susceptibility was tested using spot assay, minimal inhibitory concentration (MIC), and filter disc assay as described below.

### 2.3. Spot Assay

Spot assays for the strains were determined using a method as described elsewhere [9, 10]. Briefly, for the spot assay 5 *μ*L of fivefold serial dilutions of each yeast culture (each with cells suspended in normal saline to an OD_600 _nm of 0.1) was spotted onto YEPD plates in the absence (control) and presence of the drugs. Growth was not affected by the presence of solvent (methanol/water 2 : 1) used in the examination (data not shown). Growth difference was measured after incubation at 30°C for 48 hours. The concentrations used in this study are specified in figure legends.

### 2.4. Minimum Inhibitory Concentration (MIC)

MIC was determined by broth dilution method as described in method M27-A3 from the Clinical and Laboratory Standards Institute (CLSI) formerly NCCLS [11]. Briefly, 100 *μ*L of media was placed at each well of the 96-well plate following the addition of the drug with the remaining media and then was serially diluted. 100 *μ*L of cell suspension (in normal saline to an OD_600_ 0.1) was added to each well of the plate and OD_600_ was measured after 48 hours at 30°C. The MIC_80_ was defined as the concentration at which the 80% of the growth was inhibited.

### 2.5. Filter Disc Assay

The filter disc assay was performed as described elsewhere [9, 10, 12]. The drugs were spotted in a volume of 5–10 *μ*L at the indicated amount in the figure legends and the diameters of the respective zones of inhibition were measured after incubation of the plates for 48 hours at 30°C.

### 2.6. Rhodamine 6G Efflux

The efflux of R6G was determined by using protocol described elsewhere [13]. Briefly, approximately 1 × 10^6^ yeast cells from an overnight-grown culture in the absence (control) and presence of Ses at its subinhibitory concentration (0.552 mg/mL) determined by the growth curve experiments (data not shown) were transferred to YEPD medium and allowed to grow for 5 h. Cells were pelleted, washed twice with phosphate-buffered saline (PBS) (without glucose), and resuspended as a 2% cell suspension, which corresponds to 10^8^ cells (w/v) in PBS without glucose. The cells were then deenergized for 1 h in 2-DOG (5 mM) and 2,4 DNP (5 mM) in PBS (without glucose). The deenergized cells were pelleted, washed, and then resuspended as a 2% cell suspension (w/v) in PBS without glucose, to which R6G was added at a final concentration of 10 *μ*M and incubated for 40 min at 30°C. The equilibrated cells with R6G were then washed and resuspended as a 2% cell suspension (w/v) in PBS without glucose. Samples with a volume of 1 mL were withdrawn at the indicated time and centrifuged at 10,000 ×g for 1 min. The supernatant was collected, and absorption was measured at 527 nm. Energy dependent efflux (at the indicated time) was measured after the addition of glucose (2%) to the cells resuspended in PBS (without glucose). Glucose-free controls were included in all the experiments.

### 2.7. Passive Diffusion of Drug

The passive diffusion of R6G was determined using protocol described elsewhere [12]. Briefly, the cells cultured overnight at 30°C in the absence (control) and presence of Ses at its subinhibitory concentration were harvested, washed, and resuspended in 2% cell suspension with PBS containing 5 mM 2-DOG and 5 mM 2,4 DNP for 1 h to deenergize the cells. After that, the deenergized cells were harvested by centrifugation at 5000 g rpm for 3 min. The cells were then pelleted, washed, and resuspended in PBS 2% (w/v) with the fluorescent compound 10 *μ*M R6G for 40 min and then the cells were harvested at 5000 g rpm for 3 min, washed, and mixed with PBS (w/o glucose). The cells were then centrifuged at 10,000 g rpm for 1 min and OD_527_ of the supernatant from 0 min up until 45 min was measured at the indicated time points in Figure 3.

### 2.8. Phenotypic Susceptibility Assays

Phenotypic susceptibilities were measured using spot assays as described above. The following stock solutions were used (the solvents used are given in parenthesis): SDS, 10% w/v (water), NaCl 5 M (water), LiCl 5 M (water), CaCl_2_ 5 M (water), and DTT 1 M (water). The final chemical concentrations used for this study are specified below. Cells were spotted on YEPD plates in the absence (control) and presence of the Ses at its subinhibitory concentration and the chemicals at the following concentrations: alkaline pH 10.0, SDS (0.02% w/v), NaCl (1 M), LiCl (0.4 M) and CaCl_2_ (0.3 M), DTT (20 mM), and serum (50% v/v). For alkaline pH, YEPD plates buffered with 155 mM of Tris-Cl at pH 8.0 and 10 were used. Growth differences were recorded following incubation of the plates for 48 hours at 30°C.

### 2.9. Quantitation of Ergosterol

Sterols were extracted by the alcoholic KOH method and the percentage of ergosterol was calculated as described previously [14, 15]. Briefly, a single* C. albicans *colony from an overnight YPD agar plate culture was used to inoculate 50 mL of YPD in presence and absence of Ses. The cultures were incubated for 16 h with shaking at 30°C. The stationary-phase cells were harvested by centrifugation at 2,700 rpm for 5 min and washed once with sterile distilled water. The net wet weight of the cell pellet was determined to which 3 mL of freshly prepared 25% alcoholic potassium hydroxide solution (25 g of KOH and 35 mL of sterile distilled water, brought to 100 mL with 100% ethanol) was added to each pellet and vortex mixed for 1 min. Cell suspensions were transferred to sterile borosilicate glass screw-cap tubes and were incubated in an 85°C water bath for 1 h. Following incubation, tubes were allowed to cool to room temperature. Sterols were then extracted by addition of a mixture of 1 mL of sterile distilled water and 3 mL of* n*-heptane followed by vigorous vortex mixing for 3 min. The heptane layer was transferred to a clean borosilicate glass screw-cap tube and stored at −20°C. Both ergosterol and 24(28)-DHE absorbs at 281.5 nm, whereas only 24(28)-DHE absorb at 230 nm. Ergosterol content is determined by subtracting the amount of 24(28)-DHE (calculated from the OD_230_) from the total ergosterol plus 24(28)-DHE content (calculated from the OD_281.5_). Ergosterol content was calculated as a percentage of the wet weight of the cells with the following equations: % ergosterol + % 24(28)-DHE = [(A281.5/290) × *F*]/pellet weight; % 24(28)-DHE = [(A230/518) × *F*]/pellet weight and % ergosterol = [% ergosterol + % 24(28) DHE] − % 24(28) DHE, where *F* is the factor for dilution in petroleum ether and 290 and 518 are the *E* values (in percent per centimeter) determined for crystalline ergosterol and 24(28)-DHE, respectively.

### 2.10. Electron Microscopy of* Candida* Cells

Cells treated with Ses at its MIC_80_ value were observed by using SEM (Zeiss EVOMA10) and TEM (JEOL JEM-1011). The cells (~10^6^ cells) were administered to the media with and without Ses and were incubated for 24 h at 30°C. Sample preparation and analysis were performed by using the method as described elsewhere [16, 17]. Briefly, all cells were fixed with 2% glutaraldehyde in 0.1% phosphate buffer for 1 h at room temperature (20°C), washed with 0.1 M phosphate buffer (pH 7.2), and postfixed with 1% OsO_4_ in 0.1 M phosphate buffer for 1 h at 4°C. Then the cells were dehydrated in acetone, dropped on round glass coverslip with hexamethyldisilazane (HMDS), dried at room temperature, and then sputter coated with gold and observed under the SEM (Zeiss EVOMA10) at 30 K magnificationand TEM (JEOL JEM-1011) at 10 K magnification.

## 3. Results

### 3.1. Ses Acts as Effective Antifungal against* C. albicans *and Non-*albicans* Species

**Figure 1:**
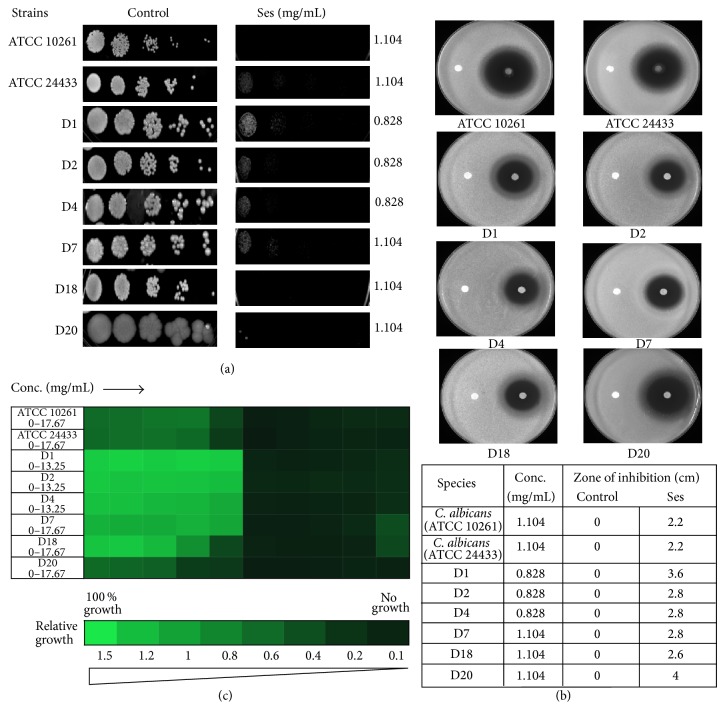
Drug susceptibility assays against* C. albicans *in the presence of Ses. (a) Spot assay of* C. albicans *reference strains (ATCC 10261, ATCC 24433) and clinical isolates (D1, D2, D4, D7, D18, and D20) in the absence (control) and presence of Ses. (b) Disc diffusion assay against* C*.* albicans* reference strains (ATCC 10261, ATCC 24433) and clinical isolates (D1, D2, D4, D7, D18, and D20) of* C. albicans *and their respective zone of inhibitions in the absence (control) and presence of Ses. For control, discs were spotted with the solvent of Ses described in Section 2. (c) Broth microdilution assay to determine the MIC_80_ of* C. albicans *reference strains (ATCC 10261, ATCC 24433) and clinical isolates (D1, D2, D4, D7, D18, and D20) in presence of Ses. Data was quantitatively displayed with colour (see colour bar), where each shade of colour represents relative optical densities of the cell. The minimum drug concentration that inhibits growth by 80% relative to the drug-free growth control is indicated as MIC_80_ for each strain.

**Figure 2:**
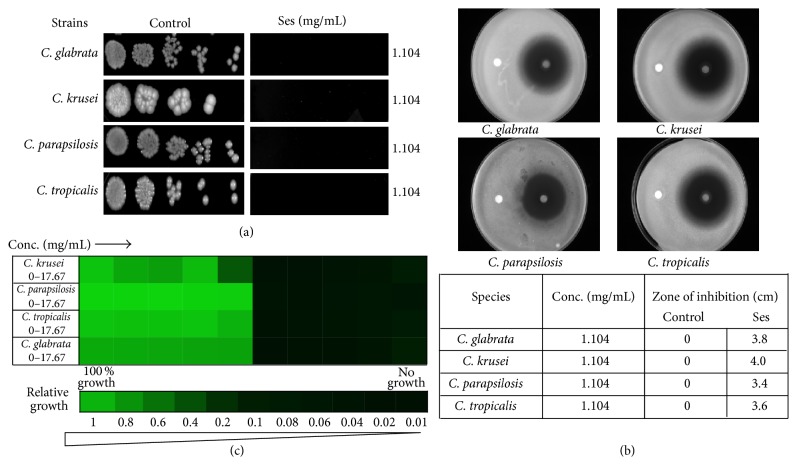
Drug susceptibility assays against non-*albicans *species of* Candida *in the presence of Ses. (a) Spot assay of* C. glabrata, C. krusei*,* C. parapsilosis,* and* C. tropicalis*, in the absence (control) and presence of Ses. (b) Disc diffusion assay against* C. glabrata, C. krusei*,* C. parapsilosis*, and* C. tropicalis*, and their respective zone of inhibitions in the absence (control) and presence of Ses. For control, discs were spotted with the solvent of Ses described in Section 2. (c) Broth microdilution assay to determine the MIC_80_ of* C. glabrata, C. krusei*,* C. parapsilosis,* and* C. tropicalis* in presence of Ses. Data was quantitatively displayed with colour (see colour bar), where each shade of colour represents relative optical densities of the cell. The minimum drug concentration that inhibits growth by 80% relative to the drug-free growth control is indicated as MIC_80_ for each strain.

To analyze the effect of Ses against* C. albicans* we firstly verify its antifungal activity by drug susceptibility testing by several autonomous methods, namely, spot assay, disc diffusion assay, and determining minimum inhibitory concentration (MIC_80_) through broth microdilution assay. Through susceptibility testing it was confirmed that Ses was showing the antifungal activity against* C. albicans*. Through both spot assay (Figure 1(a)) and filter disc assay (Figure 1(b)) it was observed that Ses was inhibiting the growth of* C. albicans *(ATCC10261, ATCC24433) at 1.104 mg/mL concentration. Broth microdilution assay also corresponds to the above results and depicts the antifungal effect of Ses (Figure 1(c)). To further test the efficacy of Ses we also performed these tests and estimated the susceptibility of Ses on six different clinical isolates of* C. albicans* (D1, D2, D4, D7, D18, and D20). We observed that the inhibitory concentration of Ses lies in the range of 0.828–1.104 mg/mL. To further elaborate our study, we extend our drug susceptibility testing against non-*albicans* species as well, namely,* C. krusei, C. tropicalis, C. parapsilosis, *and* C. glabrata*. We found that Ses was equally effective against all the tested species of* Candida* as depicted by spot, filter, and broth microdilution assays (Figures 2(a), 2(b), and 2(c)). Thus, all the drug susceptibility testing results indicate that Ses is inhibitory against reference and clinical isolates of* C. albicans *as well as non-*albicans* species (Table 1).

### 3.2. Antifungal Activity of Ses Is Independent of Multidrug Efflux Transporter Activity

**Figure 3:**
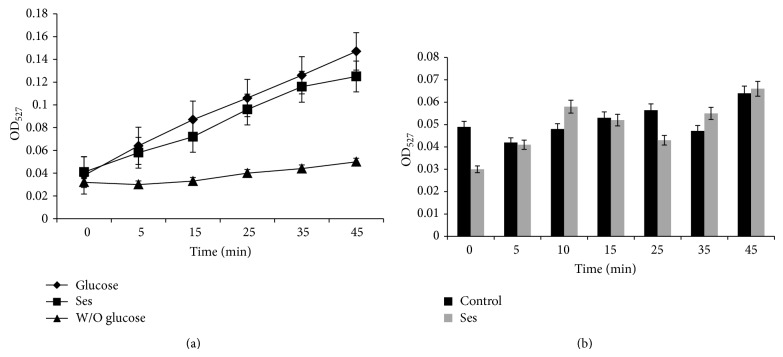
Drug efflux assay and passive diffusion in presence of Ses. (a) Extracellular concentrations of R6G for* C. albicans *(ATCC 10261) cells grown in absence (control) and presence of Ses (0.552 mg/mL) calculated as described in material and methods. Negative control represents* C. albicans* deenergized cells without glucose. Mean of OD_527_  ± SD of three independent sets of experiments are depicted on *y*-axis with respect to time (minutes) on *x*-axis. (*P* value > 0.05). (b) Passive diffusion of R6G in absence (control) and presence of Ses (0.552 mg/mL) calculated as described in Section 2. Mean of OD_527_  ± SD of three independent sets of experiments are depicted on *Y*-axis with respect to time (minutes) on *x*-axis (*P* value > 0.05).

Hyperactive efflux pumps are one of the major mechanisms responsible for the development multidrug resistance in the* Candida* [6]. To verify whether the antifungal activity of Ses is due to hindrance in efflux pumps activity or not, R6G (known substrate of efflux pumps) efflux assay was performed in presence and absence (control) of Ses. Our data confirmed that there was no significant difference (*P* value > 0.5) in the R6G efflux estimated by extracellular R6G concentration in the absence or presence of Ses (Figure 3(a)). We further accessed whether antifungal activity of Ses could be attributed to enhanced passive diffusion. Our results depict that there was no significant difference (*P* value > 0.5) in the passive diffusion of Ses in comparison to the control cells without Ses treatment (Figure 3(b)). Thus, the effect of Ses on* C. albicans* was independent of the efflux pumps activity and linked with any other pathway.

### 3.3. *C. albicans *Was Hypersensitive to Ses at Alkaline pH

**Figure 4:**
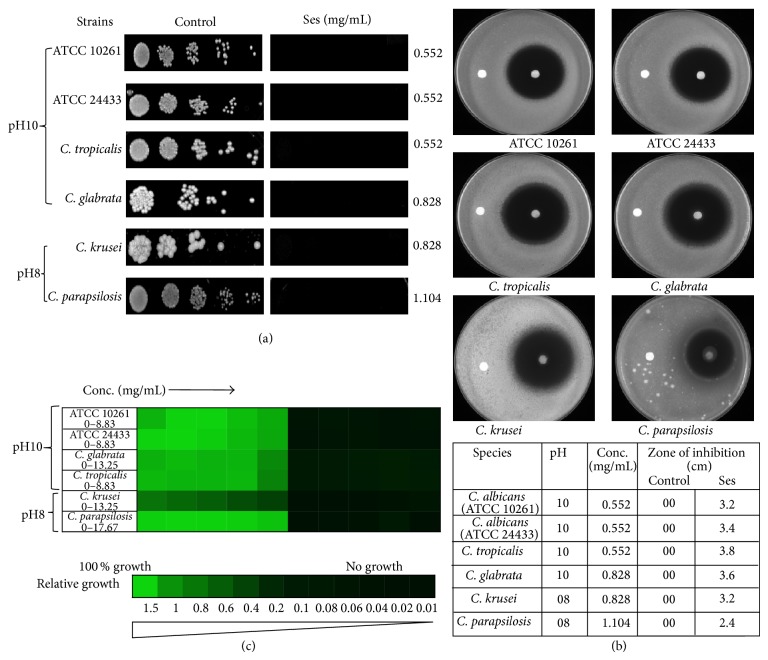
Drug susceptibility assay against* C. albicans* and non-*albicans *speciesat alkaline pH. (a) Spot assay of* C. albicans*,* C. tropicalis*, and* C. glabrata *at pH 10 and* C. krusei *and* C. parapsilosis* at pH 8 in the absence (control) and presence of Ses. (b) Disc diffusion assay against* C. albicans*,* C. tropicalis*, and* C. glabrata* at pH 10 and* C. krusei *and* C. parapsilosis* at pH 8 and their respective zone of inhibitions in the presence of Ses. For control discs were spotted with the solvent of Ses described in Section 2. (c) Broth microdilution assay to determine the MIC_80_ at alkaline pH of* C. albicans*,* C. tropicalis*, and* C. glabrata* at pH 10 and* C. krusei *and* C. parapsilosis* at pH 8 in the presence of Ses. Data was quantitatively displayed with colour (see colour bar), where each shade of colour represents relative optical densities of the cell. The minimum drug concentration that inhibits growth by 80% relative to the drug-free growth control is indicated as MIC_80_ for each strain.

*C. albicans *is an opportunistic fungus that resides in different parts of the body having varying niches. Due to this, it has an imperative ability to overcome such environmental cues. One such significant factor that* C. albicans* needs to cope with is alkaline pH. This exigent activity of* Candida* spp. compelled us to study the antifungal effect of Ses at alkaline pH range for* C. albicans* as well as non-*albicans*. It was observed that the antifungal activity of Ses was upraised to the level of almost 50% against* C. albicans* and non-*albicans *species when spotted at elevated pH as demonstrated in Figure 4(a). The results were unfaltering with the broth microdilution and disc diffusion assay as well as illustrated in Figures 4(b) and 4(c). All of these results prove that the* C. albicans *as well as non*-albicans* species of* Candida *becomes hypersensitive to Ses at alkaline pH (Table 2).

### 3.4. Ses Phenocopies Compromised Calcineurin Signaling Pathway in* C. albicans*

**Figure 5:**
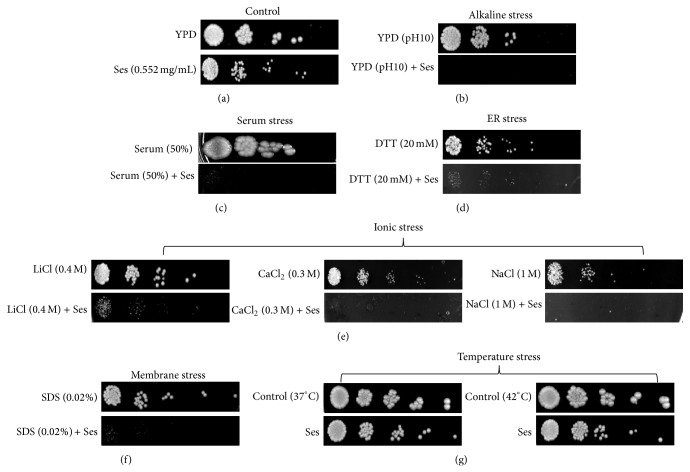
Phenotypic susceptibility assays to reveal the effect of Ses on the calcineurin dependent phenotypes in* C. albicans*. (a) Spot assay with and without Ses (0.552 mg/mL) as controls. (b) Spot assay with Ses at elevated pH 10 showing hypersensitivity to* C. albicans*. (c) Spot assay depicting the loss of growth in cells treated with serum (50% w/v) along with Ses. (d) Spot assay showing loss of growth under ER stress by DTT (20 mM) in presence of Ses. (e) Spot assays showing sensitivity in the presence of Ses under various ionic stress conditions with LiCl (0.4 M), CaCl_2 _(0.3 M), and NaCl (1 M). (f) Spot assay demonstrating the attenuated growth of* C. albicans *in the presence of Ses with membrane perturbing agent SDS (0.02% w/v). (g) Spot assay demonstrating no growth loss at elevated temperature at 37°C and 42°C in the presence of Ses.

Our above results showing hypersensitive response of* C. albicans* at alkaline pH in presence of Ses prompted us to further study the other phenotypes which were known to be governed by calcineurin signaling. Like alkaline pH response which is mediated through the calcineurin signaling cascade, we tested the remaining phenotypic response such as serum stress, ER stress, ionic stress, and membrane stress in presence of Ses. For this, we performed phenotypic susceptibility tests in the absence (control) and presence of Ses under the conditions which require functional calcineurin signaling pathway. We observed that unlike control cells (Figure 5(a)) and similar to the alkaline pH (Figure 5(b)),* C. albicans *was unable to grow when treated with 50% serum along with the Ses (Figure 5(c)). Similar hypersensitive response to Ses was observed when treated with the DTT that is known to provide ER stress to the cells (Figure 5(d)). Next we study the salinity stress response and we observed that* C. albicans* was also unable to grow in presence of Ses when treated with the elevated doses of various salts, namely, Na^+^, Ca^++^, and Li^+^ providing ionic stress to the cells (Figure 5(e)). Furthermore, the membrane stress response was tested by treating* C. albicans *with SDS and we observe a similar hypersensitivity in presence of Ses (Figure 5(f)). Notably, the cells in presence of Ses were showing resistance at elevated temperatures of 37 and 42°C and were efficiently growing (Figure 5(g)). Thus, our results demonstrate that Ses could possibly cause hindrance in the calcineurin signaling pathway in* C. albicans*.

### 3.5. Ses Causes Membrane Disruption in* C. albicans*

**Figure 6:**
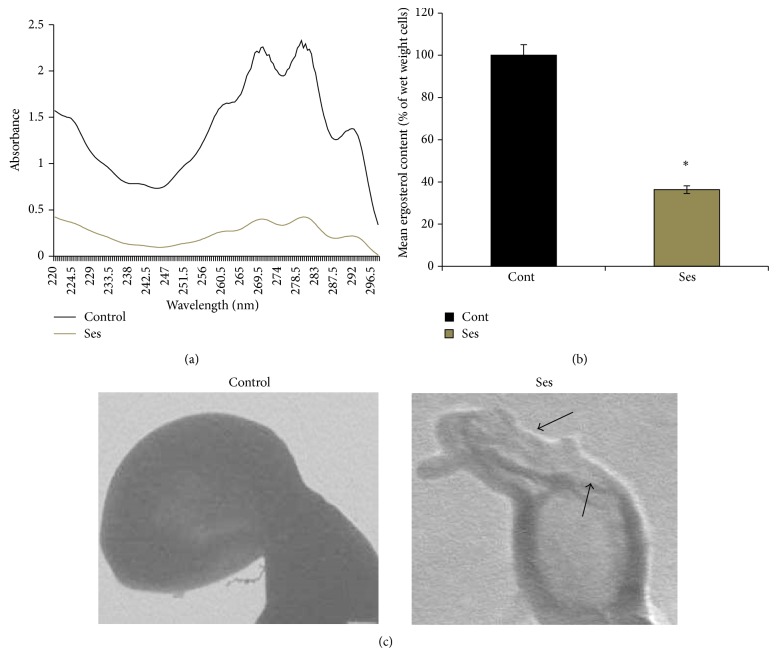
Effect of Ses on membrane composition. (a) UV spectrophotometric sterol profile of* C. albicans* scanned between 220 and 300 nm from a culture grown for 16 hours with and without Ses (0.552 mg/mL). (b) Relative percentages of ergosterol content in the absence (control) and presence of Ses (0.552 mg/mL). Mean of % ergosterol levels is calculated as described in Section 2 normalized by considering the untreated control as 100 (absolute value of 0.003) ± SD of three independent sets of experiments are depicted on *y*-axis and ∗ depicts *P* value < 0.05. (c) Transmission electron micrographic image showing the tampered membrane integrity of* C. albicans* with or without Ses as described in Section 2.

Hypersensitivity of* C. albicans* to Ses under the membrane stress, namely, SDS, necessitated further exploring the effect of Ses on membrane composition. For this, we estimated the ergosterol level which is the main component of the fungal cell membrane in the absence and presence of Ses. Interestingly, the* Candida* cells treated with Ses show dwindling in the ergosterol content to a significant level (*P* value ≤ 0.05) by around 63% (Figures 6(a) and 6(b)). Furthermore, we assessed the micrographic images through transmission electron microscopy (TEM) that also confirmed the membrane disruption because of Ses treatment (Figure 6(c)).

### 3.6. Ses Does Not Cross Talk with Cell Wall Integrity Pathway in* C. albicans*

**Figure 7:**
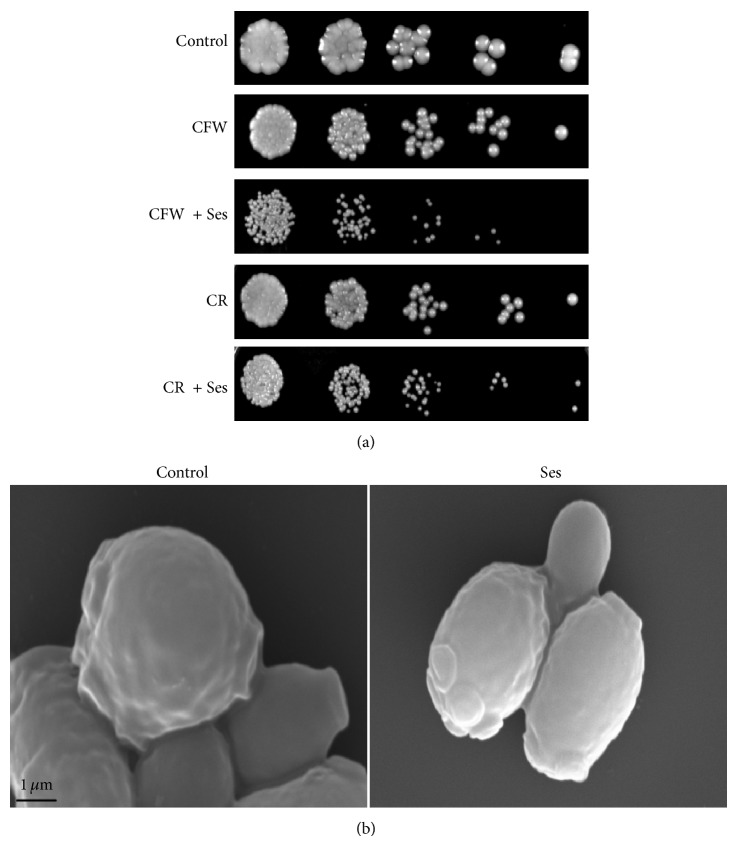
Effect of Ses on the cell wall integrity of* C. albicans*. (a) Spot assay showing no effect to Ses (0.552 mg/mL) in the presence of cell wall perturbing agents; CFW (10 *μ*g/mL) and CR (10 *μ*g/mL). (b) Scanning electron micrographs, as described in Section 2, showing the smooth surface of untreated cells (control) and treated cells.

Cell wall integrity pathway (CWI) has been linked to calcineurin signaling pathway for having implications in governing responses toward survival of exposure to antifungal drugs [18]. To test whether Ses is affecting the cell wall integrity, we performed spot assays in presence of cell wall disrupting agents, namely, CFW and CR. Our results revealed that Ses was unable to affect the cell wall silhouette when administered along with the cell wall disrupting agents and the cell growth was not affected (Figure 7(a)). The fact that Ses does not cause any cell mutilating effect was further confirmed by SEM experiments as described in Materials and Methods (Figure 7(b)). These results confirm that the anticandidal effect of Ses could not be associated with the corrugation of its cell surface morphology.

## 4. Discussion

Ses, the major constituent of sesame seed oil obtained from* Sesamum *sp., is a traditionally used supplement drug having antioxidant, anti-inflammatory, immunomodulatory, neuroprotective, antiaging, chemopreventive, proapoptotic, and antidepressant effects [19, 20]. Recently, it was shown that Ses also suppresses the effect of cyclooxygenase-2 transcriptional activity in colon cancer cells [21] which can be an important therapeutic intervention for cancer patients. The toxicological effects of sesamol have already been studied by Ambrose et al. [22] and found to be nontoxic and nonirritant and even do not cause skin sensitization. Despite the fact that Ses possesses such diverse ranges of properties for human benefits mentioned above, no direct study on its antifungal activity has been demonstrated. Hence, in this study we explore the antifungal potential and the mode of action of Ses against human fungal pathogen,* C. albicans*. We proposed that the effect of Ses against* C. albicans* might be mediated through stalled calcineurin signaling pathway leading to distorted membrane composition via ergosterol depletion. However, the antifungal effect of Ses is not affected by the multidrug efflux transporter or passive diffusion through membrane which are somewhat well-known mechanisms of action. Most of these mechanisms are critical for the virulence of* C. albicans* to survive within the host body [17, 23, 24].

Firstly, we evaluated the antifungal activity of Ses through drug susceptibility testing; spot assay, disc diffusion assay, and MIC_80_. Interestingly, we found that the growth of not only* C. albicans *but also non-*albicans* species of* Candida* was affected when grown in presence of Ses (Figures 1 and 2). These results prompted us to further examine the possible mode of action of Ses. Since the overexpression of drug efflux pumps is among the most common mechanisms responsible for the drug resistance in most of the fungi [6, 25], we checked if Ses is targeting MDR efflux transporter and passive diffusion in* C. albicans *through R6G efflux assay. It was observed that the efflux as well as passive diffusion of R6G remains unaffected in presence of Ses in* C. albicans* (Figure 3). These results assured us that the efficacy of Ses is not linked with these activities but rather have a distinct mode of action. So, we further investigated other pathways by which it could affect the growth of* C. albicans*. One of the most important factors responsible for the virulence and survival of* C. albicans* inside the host body is its survival in varying pH ranges [26, 27]. So the drug susceptibility testing of* C. albicans* at elevated pH in presence of Ses was performed and found that* C. albicans *as well as non-*albicans* species became hypersensitive to Ses at elevated pH. Interestingly, hypersensitivity was observed at much lower concentrations than the ones used at physiological pH as shown in Figure 4. It is noteworthy that, for* C. krusei* and* C. parapsilosis,* the alkaline pH response was studied at pH 8.0 since they were already hypersensitive at pH 10 and we could not differentiate any further differences in sensitivity due to Ses (data not shown). Notably, unlike* C. krusei* which was hypersensitive at pH 8.0, sensitivity of* C. parapsilosis *remained unaffected in presence of Ses. The hypersensitive response of* C*.* albicans* in presence of Ses provided us with the inkling that antifungal mechanism of Ses has some relation with calcineurin signaling cascade as it is well known that alkaline pH is one of the several phenotypes governed via calcineurin signaling pathway [28–30].

Calcineurin pathway is an effective drug target which acts through Ca^2+^/calmodulin activation based Ser/Thr dependent signaling pathway and is responsible for the virulence in* C. albicans*. Calcineurin is a dimeric protein that has remained highly conserved throughout the evolution in eukaryotes. Calcineurin includes a catalytic subunit CnA and a regulatory subunit CnB. Calcineurin activates the Crz1p, a transcription factor [31], which interacts with nuclear localization signal (NLS) Nmd5p and translocates within the nucleus and encodes the products that help in the survival of the yeast during stress conditions [32] such as alkaline pH stress, ionic stress, membrane stress, endoplasmic reticulum stress, and serum stress [33]. Hypersensitive response of* C. albicans* towards Ses at alkaline pH and its association with calcineurin signaling pathway obligated us to analyze profundity the other phenotypes which are related to the functional calcineurin pathway.

To confirm our hypothesis that the antifungal effect of Ses is because of the compromised calcineurin pathway,* C. albicans* was administered with different stress conditions in presence of Ses that crucially requires functional calcineurin cascade. Some of the stresses other than alkaline pH are serum stress, ER stress, ionic stress, membrane stress, and thermal stress. As a known fact, after invading the host body, first situation that* C. albicans *faces is the encounter with blood. Blankenship and Heitman [34] have already reported that the serum component of the blood causes the stress and is toxic to* C. albicans*. This stress is efficiently survived by the functional calcineurin signaling pathway [1]. Therefore, we study the effect of Ses on* C. albicans* in presence of serum and observed the attenuated growth of* Candida *cells at 50% concentration serum with Ses (Figure 5(c)). Likewise, ER stress is another parameter that leads to the activation of calcineurin signaling pathway by accumulating the unfolded proteins through the unfolded protein response (UPR) in eukaryotic cells [35]. Lately, Miyazaki and Kohno [36] have reported that UPR is also responsible for virulence which consequently activates calcineurin pathway in* C. albicans* but not in* C. glabrata* that lacks the fungal UPR and possesses a typical and unique alternate mechanism to evade ER stress. To observe the ER stress response,* Candida* cells were grown in the presence of DTT which could stimulate the formation of UPR by hindering N-linked glycosylation of secreted protein and inhibit the formation of disulfide bond in ER [37]. We found that* Candida* cells were hypersensitive in presence of ER stress as shown in Figure 5(d). Ionic stress response is reportedly another parameter by which we can study the calcineurin pathway [35]. We found that the ions, namely, CaCl_2_, LiCl, and NaCl, in presence of Ses checked the growth of* C. albicans* as illustrated in Figure 5(e). Similarly, it was found that* Candida* cell become hypersensitive when grown with membrane perturbing agent SDS in presence of Ses as in Figure 5(f). Another important factor that governs the activation of calcineurin signaling cascade in* Candida *as well as in other fungi is the thermotolerance. Interestingly, thermotolerance is not required for the activation of calcineurin in* C. albicans* but there are reports that prove its effects on the calcineurin in several other fungi as well as* Candida* sp. [24]. Hence, we assumed that there will be no effect of Ses on the elevated temperature stress at 37 and 42°C on* C. albicans*. As expected it was observed that irrespective of the presence of Ses,* C. albicans *cells were efficiently growing at higher temperature (Figure 5(g)). This data confirms the fact that thermal stress response is not governed by calcineurin signaling in* C. albicans* and Ses being primarily targeting the calcineurin signaling also has no influence on thermotolerance.

Perturbation of membrane structure and function was further evident from the fact that ergosterol levels were considerably reduced by around 63% in* C. albicans* when grown in presence of Ses (Figures 6(a) and 6(b)) which proves that Ses obliterates membrane veracity. This data is consistent with the fact that* C*.* albicans* cells were hypersensitive in presence of Ses when grown with membrane perturbing agent, namely, SDS (shown above). The disrupted membrane integrity of* C. albicans* in presence of Ses as also confirmed by TEM images (Figure 6(c)) clearly establishes the fact that Ses has destructive effect on membrane which could be possibly due to hindered calcineurin signaling pathway; however, further work is needed for confirmation.

Existence of evidence suggesting a crosstalk between calcineurin signaling cascade and cell wall integrity pathway [17, 23] and functional indispensability of calcineurin signaling cascade to sustain Ses exposure as demonstrated through this study necessitated to investigate the effect of Ses on cell wall damage. Intriguingly, there was no effect of Ses on cell wall damage when the cells were grown with the Ses along with cell wall perturbing agents, namely, CFW and CR (Figure 7(a)). This was further confirmed by performing SEM which shows the smooth surfaced cell wall when grown with or without Ses (Figure 7(b)). Thus, it could be said that the effective pathway by which Ses slays* C. albicans* is the disruption of cell wall integrity.

## 5. Conclusion

Despite the availability of various classes of antifungal drugs with enhanced activities, developments of drug resistance still remain a major concern for* C*.* albicans*. Thus, opting for an alternative therapeutic strategy by reverting towards natural compounds that can be used as antifungal agents could be a wise option. The data generated through this study clearly establishes the antifungal nature of Ses that can be exploited for improving the therapeutic strategies. Furthermore, these natural compounds could also hold promise to act as chemosensitizing agents that can be beneficial in reducing the dosages of current antifungal regimes.

## Figures and Tables

**Table 1:** MIC_80_ of *C. albicans* and non-*albicans* species in presence of Ses.

Strains	MIC_80_ (mg/mL)
ATCC10261	1.104
ATCC24433	1.104
D1	0.828
D2	0.828
D4	0.828
D7	1.104
D18	1.104
D20	1.104
*C. krusei *	1.104
*C. glabrata *	1.104
*C. parapsilosis *	1.104
*C. tropicalis *	1.104

**Table 2:** MIC_80_ of *C. albicans* and non-*albicans* in presence of Ses at alkaline pH.

Strains	pH	MIC_80_ (mg/mL)
ATCC10261	10	0.552
ATCC24433	10	0.552
*C. krusei *	8	0.828
*C. glabrata *	10	0.828
*C. parapsilosis *	8	1.104
*C. tropicalis *	10	0.552

## Abbreviations

Ses: Sesamol
R6G: Rhodamine 6G
2-DOG: 2-Deoxy D-glucose
2,4 DNP: 2,4-Dinitrophenol
CFW: Calcofluor white
CR: Congo red
YEPD: Yeast extract peptone dextrose
TEM: Transmission electron microscopy
SEM: Scanning electron microscopy.
